# Correlation of low fetal fraction of cell-free DNA at the early second-trimester and pregnancy complications related to placental dysfunction in twin pregnancy

**DOI:** 10.3389/fmed.2022.1011366

**Published:** 2022-12-01

**Authors:** Jiaxin Li, Xunke Gu, Yuan Wei, Yuan Tao, Bingbing Zhai, Chunfang Peng, Quanfei Huang, Tao Deng, Pengbo Yuan

**Affiliations:** ^1^Department of Obstetrics and Gynecology, Peking University Third Hospital, Beijing, China; ^2^CapitalBio Medical Laboratory, Beijing, China; ^3^CapitalBio Technology Co., Ltd., Beijing, China

**Keywords:** cell-free DNA, fetal fraction, twin pregnancy, pregnancy complications, early screening

## Abstract

**Introduction:**

This study aimed to determine the correlation between fetal fraction (FF) of cell-free DNA (cf-DNA) and pregnancy complications related to placental dysfunction in Twin Pregnancy.

**Methods:**

This retrospective cohort study analyzed twin pregnant women who underwent non-invasive prenatal testing (NIPT) at 12^+0^–26^+6^ weeks of gestation from April 2017 to April 2021. Low fetal fraction (LFF) was defined individually as less than the 25th, 10th, 5th, and 2.5th percentile among all fetal fractions in the cohort. Primary outcomes included gestational hypertension (GH), preeclampsia (PE), gestational diabetes mellitus (GDM), and small for gestational age (SGA). Logistic regression analysis was used to assess the relationship between LFF and pregnancy complications.

**Results:**

A total of 500 twin pregnancies (male-male twins, 245; female-female twins, 255) were included in this study. In LFF group (FF < 25th percentiles), maternal BMI was significantly higher than FF > 75th percentiles (23.6 kg/m^2^ vs. 21.3 kg/m^2^; *P* < 0.001). The risk of SGA increased gradually from FF < 25th percentiles [adjusted odds ratio (OR), 1.71; 95% confidence interval (CI), 1.07–2.99; *P* = 0.016] to FF < 2.5th percentiles (adjusted OR, 4.44; 95% CI,1.33–14.82; *P* < 0.015). In addition, the risks of SGA in both fetuses were higher than the risks of at least one fetus SGA in LFF group. LFF had no correlation with GH, PE, and GDM in twin pregnancy.

**Conclusion:**

LFF has a strong association with increased risk of SGA in twin pregnancy. Moreover, FF of cf-DNA may provide a new idea for the early screening of diseases related to placental dysfunction in twin pregnancy.

## Introduction

In 1997, Lo et al. (1) discovered the Y chromosome in the maternal peripheral blood of pregnant male fetuses, which proved the existence of fetal cell-free-DNA (cf-DNA) in the peripheral blood of pregnant women. In recent years, fetal cf-DNA has been widely used in fetal aneuploid screening in the early second-trimester as a biomarker for a non-invasive prenatal test (NIPT). The fetal fraction (FF) of cf-DNA is a key factor in whether NIPT can achieve effective results. In previous studies, NIPT would received no-call results when FF is <4%, which is affected by diverse maternal-fetal factors, such as gestational age, maternal weight, race, and multiple pregnancies (2–11). In addition, fetal cf-DNA is mainly derived from placenta trophoblast cell apoptosis and released into the maternal peripheral blood circulation. Therefore, FF of cf-DNA is also related to placental dysfunction, which may be a marker to the early screening for pregnancy complications with placental dysfunction, such as gestational hypertension (GH), preeclampsia (PE), small for gestational age (SGA), premature birth (PTB), and gestational diabetes mellitus (GDM).

Previous studies reported that the FF of singleton pregnant women who subsequently developed PE, GDM, PTB, and SGA were significantly different from the FF of healthy pregnant women, although the specific changes of FF were unclear. Zhong et al. suggested that the FF of PE women was higher than normal pregnant women, probably due to impaired placental function, and accelerated trophoblast apoptosis (12, 13). However, as an increasing concern of NIPT failure, several studies also found that low FF was associated with increased risks of subsequent PE, SGA, PTB, and GDM. Krishna et al. found that compared to the group with FF > 4%, the incidence of hypertension-related diseases was significantly increased in the low FF group (FF < 4%) (26.4% vs. 59.1%; *P* = 0.001) and that the low FF group had higher risks of adverse perinatal outcomes [adjusted odds ratio (OR), 2.5; 95% confidence interval (CI), 1.01–6.2; *P* = 0.049] (14). Clapp et al. determined that the proportion of low birth weight in the low FF group (FF < 5th percentile) was significantly higher than that in the FF > 5th percentile group of 2,035 singleton pregnant women (6.9% vs. 3.2%; *P* = 0.04) which may be related to placental dysplasia and placental volume decrease (15).

The above studies proved the relationship between FF and singleton perinatal outcomes, but were primarily limited by the lack of twin pregnancy cohorts. The frequency of twin gestations has increased over the last few decades, mainly due to the increased rates of advanced maternal age and the widespread use of assisted reproduction techniques. Twin pregnancy is associated with a higher risk of PE, GDM, and PTB than singleton pregnancy, which makes it necessary to find an appropriate marker for early screening of twin pregnancy complications. The NIPT landscape has been rapidly evolving in the prenatal screening of twin pregnancies. This study aimed to investigate the relationship between FF of cf-DNA and the pregnancy complications related to placental dysfunction in twin pregnancies and to determine whether FF of cf-DNA may also be used as a marker for early screening of placental dysfunction disorders in twin pregnancies.

## Materials and methods

### Study population and data collection

This retrospective cohort study analyzed all twin pregnancy women undergoing NIPT at 12–26 weeks’ gestation in Peking University Third Hospital (a university-affiliated tertiary hospital) from April 2017 to April 2021. This study was approved by the ethics committee of Peking University Third Hospital (no. M2021225) prior to the data collection. Written informed consent was obtained from each participant. The data downloaded for analysis were anonymous. We included male-male twin pregnancies and female-female twin pregnancies with the quantification of FF and pregnancy outcome information. Exclusion criteria were as follows: twin gestations with different sex; vanishing twin; fetus having chromosomal abnormalities or structural malformations; pre-gestational diseases such as chronic hypertension and diabetes; and missing or incomplete medical records. Our primary outcomes included GH, PE, GDM, and SGA. Maternal characteristics, including age, body mass index (BMI), and gestational age on NIPT, medical history, parity, method of conception, chorionicity, and FF, were obtained from our hospital’s prenatal screening database. Maternal and neonatal outcomes were obtained from our hospital’s electronic medical records, which included pregnancy complications, delivery gestations, birthweight, and sex of the two newborn babies.

### Fetal fraction measurements

Two types of methods were used to calculate the fetal DNA fraction in maternal plasma. For pregnancy with male-male fetuses, reads proportion on the Y chromosome was used to estimate the FF. For pregnancy with female-female fetuses, the FF was estimated using the length distribution of cf-DNA, in which fetal DNA (130–140 bp; region A) was generally shorter than maternal DNA (155–175 bp; region B). LOESS regression was applied to calculate the FF against reads ratio in features A and B (16). However, it was difficult to accurately calculate the FF of opposite-sex twins.

### Diagnostic measurements

According to the International Association of Diabetic Pregnancy Research Group criteria, diagnosis of GDM depended on a 75 g oral glucose tolerance test at 24–28 weeks’ gestation (17). Women with normal blood pressure who developed hypertension (systolic blood pressure ≥140 mmHg and/or diastolic blood pressure ≥90 mmHg) after 20 weeks of gestation, were diagnosed GH. PE was identified if a woman had new onset hypertension after 20 weeks of gestation, accompanied by at least one of proteinuria, other maternal organ dysfunction (including heart, lung, liver, and kidney), hematological, digestive, neurological involvement, or uteroplacental dysfunction (18). SGA was defined as the birth weight of neonate <10th percentile for the gestational age based on the global reference for birthweight percentiles (19).

### Statistical analysis

Based on the distribution of FF in the cohort, the cohort was grouped by FF quartiles to compare the baseline characteristics. The cohort was further classified into three groups to compare the relationship between FF and outcomes as follows: low FF, <2.5th, <5th, <10th, and <25th percentiles; normal FF, 2.5–97.5th, 5–95th, 10–90th, and 25–75th percentiles; and high FF, >75th, >90th, > 95th, and >97.5th percentiles.

In this study, continuous variables of baseline characteristics were non-normally distributed and expressed as median (interquartile range, IQR), whereas categorical variables of baseline characteristics and pregnancy complications were expressed as *N* (%). Kruskal–Wallis test and Chi–square test or Fisher’s exact test were used to analyze continuous and categorical variables, respectively. Multivariable logistic regressions were performed to determine OR and 95% CI to assess the association between low FF and pregnancy complications. Adjusted regressions were controlled for maternal age, BMI, and gestational age on NIPT, parity, method of conception, delivery gestations, which were selected *a priori* given their potential associations with the FF, and pregnancy complications. All data were analyzed using SPSS version 26.0. Statistical significance was set at *P* < 0.05.

## Results

**FIGURE 1:**
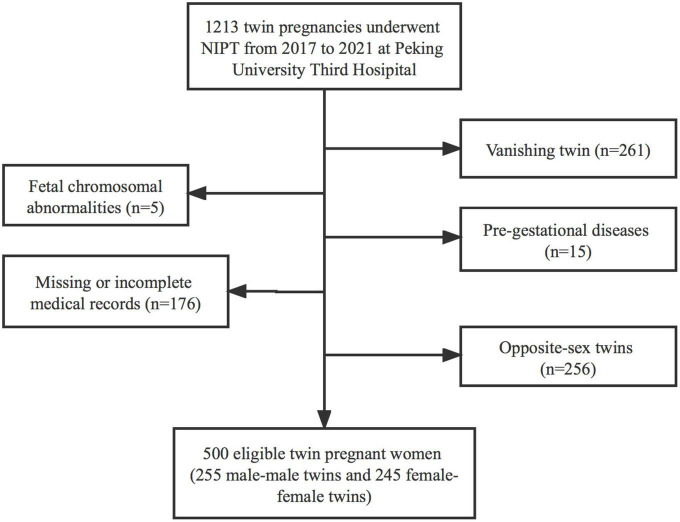
Flow chart of the study cohort.

A total of 1,213 pregnant women who underwent NIPT at 12–26 weeks of gestation after obtaining their informed consent were enrolled in this study. The following were excluded from the study: 256 women due to twin pregnancies of different sex, 261 women due to vanishing twins, and 196 observational participants due to fetus having chromosomal abnormalities (*n* = 5), pre-gestational diseases (*n* = 15), missing or incomplete medical records (*n* = 176). Finally, 500 [male-male twin pregnancies, 255 (51.0%); female-female twin pregnancies, 245 (49.0%)] were included (Figure 1).

The baseline demographic characteristics of pregnant women and obstetrical characteristics, including maternal age, BMI, and gestational age on NIPT, parity, method of conception, chorionicity, delivery gestational age, and birthweight grouped by FF quartiles were presented in Table 1. In our study, the median FF was 18.55% (IQR, 15.00–22.80%). Maternal BMI on NIPT was significantly higher in low FF (<25th percentiles) than that in other FF groups (23.6 kg/m^2^ vs. 23.2 kg/m^2^ vs. 22.3 kg/m^2^ vs. 21.3 kg/m^2^; *P* < 0.001), and the proportion of obese pregnant women was also significantly increased in low FF (10.5 vs. 7.9% vs. 4.8% vs. 4.0%; *P* < 0.001). When compared with normal FF and high FF groups, lower birthweight of the second twin was found in FF < 25th percentiles (2,290 kg vs. 2,390 vs. 2,400 vs. 2,435 kg; *P* = 0.048). However, the median maternal age (31 vs. 31 vs. 32 vs. 32 years old; *P* = 0.750) and median gestational age of NIPT (15^+0^ vs. 14^+0^ vs. 14^+0^ vs. 14^+1^ weeks; *P* = 0.184) were similar among the FF quartile groups. Similarly, the proportion of parity, method of conception, chorionicity, delivery gestational age, and birthweight of the first twin were not statistically significant among different FF percentiles groups.

**TABLE 1:** Baseline demographic characteristics of pregnant women on NIPT in the study cohort based on the categories of FF (*n* = 500).

	FF quartile (%)				*P*-value
	<25th (<15.00, *N* = 124)	25th∼50th (15.00∼18.55, *N* = 126)	50th∼75th (18.55∼22.80, *N* = 125)	>75th (>22.80, *N* = 125)	
Maternal age on NIPT (years)	31 (29∼34)	31 (29∼34)	32 (30∼34)	32 (29∼34)	0.750
Gestational age on NIPT (week)	15^+0^ (13^+1^∼16^+4^)	14^+0^ (13^+0^∼16^+2^)	14^+0^ (12^+6^∼16^+1^)	14^+1^ (13^+0^∼16^+2^)	0.184
Maternal BMI on NIPT (kg/m^2^)	23.6 (21.2∼26.5)	23.2 (21.0∼25.4)	22.3 (20.7∼24.0)	21.3 (19.8∼22.8)	**<0.001**
Obesity^a^	13 (10.5%)	10 (7.9%)	6 (4.8%)	5 (4.0%)	**<0.001**
**Method of conception**					0.833
Natural conception	78 (62.9%)	79 (62.7%)	74 (59.2%)	81 (64.8%)	
Assisted reproduction	46 (37.1%)	47 (37.3%)	51 (40.8%)	44 (35.2%)	
**Parity**					0.116
Primipara	106 (25.1%)	114 (27.0%)	103 (24.3%)	100 (23.6%)	
Multipara	18 (23.4%)	12 (15.6%)	22 (28.6%)	25 (32.5%)	
**Chorionic**					0.622
DCDA twins	61 (23.7%)	61 (23.7%)	70 (27.2%)	65 (25.3%)	
MCDA twins	63 (25.9%)	65 (26.7%)	55 (22.6%)	60 (24.7%)	
Gestational age of delivery	36^+0^ (34^+1^∼37^+0^)	36^+3^ (35^+1^∼37^+1^)	36^+3^ (34^+3^∼37^+1^)	36^+5^ (35^+2^∼37^+1^)	0.059
First twin birthweight	2,370 (1,985∼2,660)	2,440 (2,255∼2,670)	2,440 (2,100∼2,720)	2,515 (2,240∼2,690)	0.155
Second twin birthweight	2,290 (1,865∼2,615)	2,390 (2,130∼2,660)	2,400 (2,110∼2,720)	2,435 (2,182∼2,670)	**0.048**
GH	16 (12.9%)	7 (5.6%)	8 (6.4%)	6 (4.8%)	0.057
PE	29 (23.4%)	32 (25.4%)	22 (17.6%)	24 (19.2%)	0.404
GDM	29 (23.4%)	38 (30.2%)	24 (19.2%)	21 (16.8%)	0.060
**SGA**					
At least one fetus SGA^b^	29 (23.4%)	24 (19.0%)	15 (12.0%)	16 (12.8%)	**0.048**
Two fetuses SGA^c^	13 (10.5%)	4 (3.2%)	5 (4.0%)	3 (2.4%)	**0.013**

Data was presented as median (IQR) and *N* (%); ^a^maternal BMI > 28 kg/m^2^ as the standard of obesity in China; ^b^at least one fetus of twins developed small for gestational age; ^c^both fetuses of twins developed small for gestational age. FF, fetal fractions; NIPT, non-invasive prenatal test; BMI, body mass index; DCDA, dichorionic diamniotic; MCDA, monochorionic diamniotic; GH, gestational hypertension; PE, preeclampsia; GDM, gestational diabetes mellitus; SGA, small for gestational age. Bold values of *P*-values were statistically significant.

**FIGURE 2:**
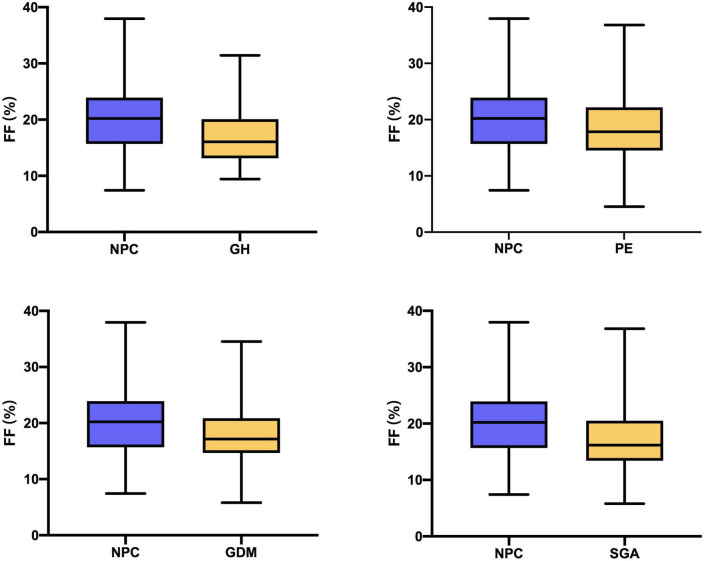
Fetal fraction (FF) in women with non-pregnancy complication (NPC) and women who developed gestational hypertension (GH), preeclampsia (PE), gestational diabetes mellitus (GDM), and small for gestational age (SGA).

The prevalence of different complications at different percentiles groups and the distribution of plasma FF in non-pregnancy complication (NPC), GH, PE, GDM, and SGA is summarized in Tables 1, 2. Among the 500 same-sex twin pregnancies, the overall prevalence of GH, PE, GDM, and SGA was 7.4% (37), 21.4% (107), 22.4% (112), and 16.8% (84), respectively. For low FF (<25th percentiles), the incidence of SGA was significantly higher than high FF (>75th percentiles) (at least one fetus SGA: 23.4% vs. 12.8%; *P* = 0.048; two fetuses SGA: 10.5% vs. 2.4%; *P* = 0.013). The rates of GH, PE and GDM in low FF (<25th percentiles) also were higher than FF 50th–75th percentiles and FF > 75th percentiles, whereas there was no significant statistical difference (GH: 12.9% vs. 6.4% vs. 4.8%; *P* = 0.057; PE: 23.4% vs. 17.6% vs. 19.2%; *P* = 0.404; GDM: 23.4% vs. 19.2% vs. 16.8%; *P* = 0.060). When compared to women with NPC, the median FF was significantly lower for women who developed GH, PE, GDM, and SGA in Figure 2 and Table 2 (NPC: 20.22% vs. GH: 16.05% vs. PE: 17.85% vs. GDM: 17.16% vs. SGA: 16.17%; all *P* < 0.01).

**TABLE 2:** The different percentiles of FF on NIPT based on pregnancy complications.

Pregnancy complications (*N*%)	2.5th (%)	5th (%)	10th (%)	25th (%)	50th (%)	75th (%)	90th (%)	95th (%)	97.5th (%)	*P-*value^a^
NPC (200, 40.0%)	10.30	11.20	12.62	15.68	20.22	23.93	27.86	31.66	34.38	
GH (37, 7.4%)	9.41	9.49	10.76	13.13	16.05	20.08	27.04	29.42	31.30	**0.008**
PE (107, 21.4%)	6.91	10.39	11.24	14.51	17.85	22.18	25.84	29.93	32.51	**0.021**
GDM (112, 22.4%)	7.63	9.35	11.09	14.67	17.16	20.87	25.66	30.93	34.08	**0.003**
SGA (84, 16.8%)	7.03	8.02	10.35	13.42	16.17	20.49	26.97	29.86	33.98	**0.001**

^a^Compared with the NPC group. FF, fetal fraction; NIPT, non-invasive prenatal test; NPC, non-pregnancy complication; GH, gestational hypertension; PE, preeclampsia; GDM, gestational diabetes mellitus; SGA, small for gestational age. Bold values of *P*-values were statistically significant.

**FIGURE 3:**
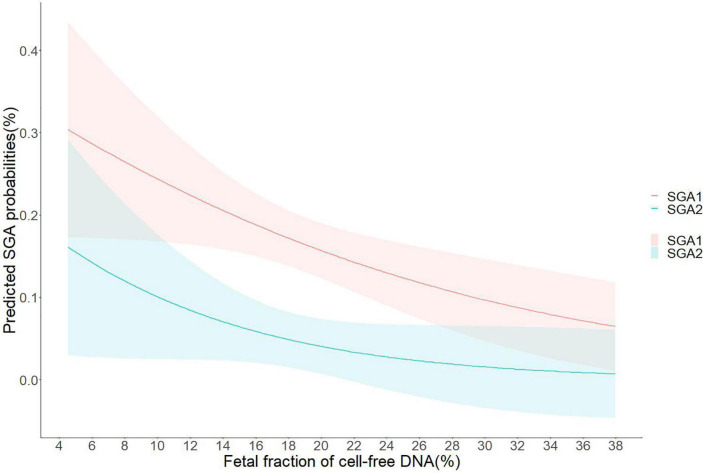
Predicted small for gestational age (SGA) probabilities with 95% CIs were calculated with respect to fetal fraction (FF) by performing logistic regression models; SGA1, at least one fetus of twins developed small for gestational age; SGA2, both fetuses of twins developed small for gestational age.

Associations of plasma FF with risk for pregnancy complications related to placental dysfunction in adjusted models were demonstrated in Table 3. In FF quartiles groups, low FF (<25th percentiles) was associated with an increased risk of SGA (at least one fetus SGA) when compared to normal FF (adjusted OR, 1.71; 95% CI, 1.07–2.99; *P* = 0.016), and high FF (adjusted OR, 2.16; 95% CI, 1.05–4.42; *P* = 0.035). We also found that low FF below the 10th, 5th, and 2.5th percentiles were associated with gradually increased risks of SGA relative to the 10th–90th, 5th–95th, and 2.5th–97.5th percentiles, respectively (for <10th vs. 10th–90th percentiles: adjusted OR, 2.99; 95% CI, 1.08–8.28; *P* = 0.018; for <5th vs. 5–95th percentiles: adjusted OR, 3.43; 95% CI, 1.39–8.47; *P* = 0.008; for <2.5th vs. 2.5th–97.5th percentiles: adjusted OR, 4.44; 95% CI, 1.33–14.82; *P* = 0.015). Furthermore, the risks of both fetuses SGA were higher in low FF than the risks of at least one fetus SGA in the corresponding FF groups (for <25th vs. 25th–75th percentiles: adjusted OR, 3.00; 95% CI, 1.22–7.39; *P* = 0.017; for <10th vs. 10th–90th percentiles: adjusted OR, 3.19; 95% CI, 1.22–8.33; *P* = 0.036; for <5th vs. 5th–95th percentiles: adjusted OR, 3.84; 95% CI, 1.14–12.91; *P* = 0.030; for <2.5th vs. 2.5th–97.5th percentiles: adjusted OR, 6.09; 95% CI, 1.69–21.95; *P* = 0.006). The associations between FF and the risk of SGA were presented in the regression curve (Figure 3). However, there was no significant association between low FF and GH, PE, and GDM.

**TABLE 3:** Association between different FF percentiles on NIPT and pregnancy complications related to placental dysfunction in adjusted models.

FF percentiles	GH		PE		GDM		SGA1		SGA2	
	OR (95% CI)	*P*	OR (95% CI)	*P*	OR (95% CI)	*P*	OR (95% CI)	*P*	OR (95% CI)	*P*
<25th	1.83 (0.64∼5.19)	0.257	1.04 (0.55∼1.97)	0.904	1.46 (0.76∼2.80)	0.258	**2.16 (1.05∼4.42)**	**0.035**	**4.41 (1.17∼16.67)**	**0.029**
25th∼50th	0.74 (0.22∼2.43)	0.614	1.26 (0.68∼2.35)	0.469	2.43 (0.93∼6.34)	0.070	1.64 (0.80∼3.38)	0.177	1.28 (0.27∼6.04)	0.753
50th∼75th	1.05 (0.34∼3.21)	0.934	0.81 (0.43∼1.56)	0.536	0.88 (0.45∼1.75)	0.723	0.42 (0.90∼1.96)	0.799	1.67 (0.39∼7.26)	0.492
>75th	1		1		1		1		1	
25th∼75th	1		1		1		1		1	
<25th	2.08 (0.94∼4.61)	0.069	1.01 (0.60∼1.70)	0.979	0.98 (0.59∼1.65)	0.951	**1.71 (1.07∼2.99)**	**0.016**	**3.00 (1.22∼7.39)**	**0.017**
>75th	1.12 (0.41∼3.10)	0.821	0.98 (0.57∼1.71)	0.949	0.69 (0.39∼1.22)	0.206	0.81 (0.42∼1.54)	0.513	0.68 (0.18∼2.59)	0.568
10th∼90th	1		1		1		1			
<10th	1.15 (0.39∼3.40)	0.800	1.56 (0.80∼3.04)	0.189	1.29 (0.64∼2.58)	0.477	**2.99 (1.08∼8.28)**	**0.018**	**3.19 (1.22∼8.33)**	**0.036**
>90th	1.27 (0.35∼4.59)	0.718	0.83 (0.37∼1.86)	0.643	0.99 (0.47∼2.11)	0.990	1.13 (0.36∼3.60)	0.831	1.03 (0.23∼4.74)	0.967
5th∼95th	1		1		1		1			
<5th	1.74 (0.45∼6.76)	0.422	1.40 (0.55∼3.52)	0.481	1.80 (0.73∼4.44)	0.201	**3.43 (1.39∼8.47)**	**0.008**	**3.84 (1.14∼12.91)**	**0.030**
>95th	0.72 (0.09∼5.81)	0.754	1.06 (0.38∼2.94)	0.915	1.27 (0.48∼3.33)	0.631	0.74 (0.21∼2.62)	0.636	1.01 (0.13∼8.07)	0.992
2.5th∼97.5th	1		1		1		1		1	
<2.5th	0.00	0.999	1.79 (0.52∼6.15)	0.358	1.87 (0.54∼6.52)	0.326	**4.44 (1.33∼14.82)**	**0.015**	**6.09 (1.69∼21.95)**	**0.006**
>97.5th	1.48 (0.18∼12.33)	0.718	1.14 (0.31∼4.20)	0.842	2.23 (0.73∼6.83)	0.161	0.83 (0.18∼3.88)	0.813	1.81 (0.21∼15.37)	0.586

FF, fetal fractions; NIPT, non-invasive prenatal test; BMI, body mass index; GH, gestational hypertension; PE, preeclampsia; GDM, gestational diabetes mellitus; SGA, small for gestational age; SGA1, at least one fetus of twins developed small for gestational age; SGA2, both fetuses of twins developed small for gestational age. Bold values of OR with 95% CIs and *P*-values were statistically significant.

## Discussion

This study demonstrates the association between low FF and pregnancy complications related to placental dysfunction of twin pregnancies. FF of twin pregnant maternal plasma cf-DNA in the first second-trimester may have the function as a marker of subsequent SGA. To the best of our knowledge, this is the first retrospective study that associated FF of twin pregnant maternal plasma cf-DNA at NIPT and subsequent risks of pregnancy complications in a Chinese population.

We demonstrated that low FF, defined individually as <25th, 10th, <5th, and <2.5th percentiles, at 12–26 weeks of gestation were strongly associated with the risk of SGA compared with the normal FF and high FF, especially the SGA of both fetuses. The result was consistent with previous publications using singleton women. Recently, several previous studies had investigated that low FF of singleton pregnancy was associated with pregnancy complications, such as PE, GDM, PTB, and SGA. Clapp et al. investigated that women with low FF of <5th (FF < 5.34%) percentile were associated with an increased risk of birth weights <5th and <10th percentiles in women with negative cf-DNA screening in the first trimester of a total of 7,478 singleton women (20). Similarly, Yuan et al. examined the significant relationship between FF < 5th percentile and low birth weight babies (<2,500 g) in 2,191 singleton women who underwent NIPT at 13–26 weeks of gestation (21). However, Stein et al. examined the relationship between second-trimester FF and SGA in 611 women who underwent NIPT and found no significant relationship (22). Our findings were similar to the findings that LFF was associated with SGA. Among twin pregnant women undergoing NIPT at our centers, the likelihood of at least one twin SGA with an LFF result (<25th percentile) is 1.71-fold higher, and this risk of SGA increased with decreasing FF (2.99-fold higher of FF < 10th percentile; 3.43-fold higher of FF < 5th percentile; and 4.44-fold higher of FF < 2.5th percentile). In addition, the risk of both fetuses SGA was higher than at least one twin SGA in LFF groups (FF < 25th percentile: 3.00-fold vs. 1.71-fold; FF < 10th percentile: 3.19-fold vs. 2.99-fold; FF < 5th percentile: 3.84-fold vs. 3.43-fold; and FF < 2.5th percentile: 6.09-fold vs. 4.44-fold). These findings may be related to the declined apoptosis of trophoblast cells induced by the smaller the fetal weight and the decreased placental volume.

Previous studies also examined the correlation between low FF and GH, PE, GDM, and PTB in a singleton pregnancy. Kristin et al. reported a strong association between low FF (<25th percentile) and hypertensive disease of pregnancy and preeclampsia with severe features (14). Chan et al. found that women who failed to obtain a result from NIPT were at increased risk of GDM compared with the general Australian obstetric population (23). Similarly, Yuan et al. provided evidence that low FF of <10th percentile was associated with increased risk of PE and early PTB (<34 weeks) in a singleton women retrospective cohort (21). Unlike these previous studies, we demonstrated that low FF was not significantly associated with GH, PE, and GDM, although the rates of GH and PE were higher in FF < 25th percentile. It might also be related to the sample size being too small to detect significant differences in these perinatal outcomes. Furthermore, our study did not include PTB as an adverse perinatal outcome in consideration of a wide range of infections, genetic, and maternal complex causes. More significantly, twin pregnancy also was an independent risk factor for PTB.

Fetal fraction was defined as the percentage of fetal origin DNA levels from maternal plasma total cf-DNA, which was generally in the range of 3–30% (9). The reliability of NIPT largely depended on the FF in the total cf-DNA of maternal plasma. If the FF was <4%, NIPT was largely unable to receive reliable results. Krishna et al. examined the association between low FF (<4%) and a composite of adverse pregnancy outcomes among a cohort of 370 women who underwent NIPT. However, no difference in the odds of low birthweight was observed between the FF < 4% and FF > 4% groups (14), which suggested that the incidence of NIPT failure due to FF < 4% was much less than that of the sufficient FF group. Our study considered the 25th percentile as the low FF cutoff value to increase the sample size and to ensure a similar sample size among quartile groups. We also set the 10th, 5th, and 2.5th percentile as the low FF cutoff values and adjusted for any available confounding factors in statistical analysis to determine the suitable FF threshold and predict pregnancy complications. Furthermore, FF was affected by multiple factors, including maternal BMI, gestational age, race, and twin pregnancy (24, 25). Our study also supported the association that decreased FF with increasing maternal BMI. In particular, we observed the significantly increased rates of obese women (BMI ≥ 28 kg/m^2^) in low FF, which suggested the increased maternal blood volume and active remodeling of adipose tissue, leading to an increased release of maternal cf-DNA into the peripheral circulation, a dilutional effect as the principal causes (26). However, we found no significant difference in the gestational age of NIPT among FF quartile groups, which may be related to the similar timing of gestational age at the obstetric examination.

Our study was novel in that we examined the relationship between low FF and pregnancy complications related to placental dysfunction among twin pregnant women who underwent NIPT in the first second-trimester. Most previous studies of the association between low FF and pregnancy complications were on singleton pregnancies. We expanded the scope of the applicable pregnancy population, including the cf-DNA test screening for pregnancy complications with placental compromise. Another strength of our study was that all twin pregnant women were admitted to and managed by our hospital, a tertiary referral care center for twin pregnancies in China, and the information regarding newborn and maternal outcomes could be recorded precisely. Moreover, the number of twin pregnancies in our center was relatively larger than in other obstetrics centers in the Beijing area, despite the fewer populations than in previous studies of singleton pregnancies.

Our study has several limitations that should be considered when interpreting our results. First, this was a retrospective study from a single institution, and FF of cf-DNA was analyzed in the same laboratory, which limited the generalizability of our findings. Multi-center prospective studies with larger twin pregnancy cohorts that would increase the generalizability are needed to further investigate FF in the first trimester using diverse laboratory technologies. In addition, longitudinal studies that analyzed the trajectory of FF over different gestational ages were also necessary to determine its predictive value for pregnancy outcomes associated with placental dysfunction. Second, although the accurate estimation of FF using length distribution of cf-DNA had been verified, we could not exclude the maternal cf-DNA that could affect FF measurements of female-female fetuses. A fairly large number of opposite-sex twins were excluded in our study, considering the difficulties in estimating and distinguishing the FF of opposite-sex twins. Finally, we use low-coverage sequencing as the routine test procedure of NIPT in this study, and zygosity information were not obtained when used low-coverage whole-genome sequencing. Analysis of single nucleotide polymorphisms (SNPs) in cf-DNA for the assessment of zygosity will be established in the follow-up study.

## Conclusion

In conclusion, twin pregnant women with low FF of maternal plasma cf-DNA in the first second-trimester are more likely to subsequently develop SGA. FF of cf-DNA may serve as a biomarker for pregnancy complications related to placental dysfunction in twin pregnancies.

## Data availability statement

The original contributions presented in this study are included in the article/supplementary material, further inquiries can be directed to the corresponding authors.

## Ethics statement

Written informed consent was obtained from the individual(s) for the publication of any potentially identifiable images or data included in this article.

## Author contributions

JL and XG completed the design and writing of the manuscript. JL, BZ, YT, CP, and QH made contributions to the acquisition, analysis, and interpretation of data. YW critically revised the work. PY and TD were accountable for all aspects of the work. All authors contributed to the article and approved the submitted version.
